# New frontiers in risk assessments related to diets, health and the environment

**DOI:** 10.1093/eurpub/ckac129.357

**Published:** 2022-10-25

**Authors:** M Springmann

**Affiliations:** Nuffield Department of Population Health, University of Oxford, Oxford, UK

## Abstract

Based on the sustainable development goals agenda, public health experts should approach the study of planetary diets, environment, and health with a holistic approach that includes both human and planetary health. Modelling studies help in estimating priorities while attaining differences across countries. Future scenarios vary according to priorities to be set. When aiming only at fulfilling food-security objectives, studies suggest that reductions in premature mortality have few environmental co-benefits. In contrast, modelling studies that include environmental goals indicate the potential for reducing environmental impacts while improving diet quality and overall health outcomes, especially in high-income countries. The scenarios indicate that focusing on dietary changes towards healthy and balanced dietary pattern seems an ideal strategy to improve both mortality risk and the environmental impact, although the magnitude of the effects may vary across countries. Weight and dietary risk factors have traditionally been considered separate categories. A new approach can be proposed for attributing weight-related risks to food intake at global, regional, and national levels. When comparing current consumption patterns to diets that for each country minimise diet and weight-related risks, a total of 5 million weight-related deaths in 2019 were attributed to the overconsumption of refined grains, dairy, red and processed meat, sugar, and eggs, and to the underconsumption of oils, and legumes, among others. A large share of deaths attributable to high intake of animal source foods and imbalanced grain intake occurred in countries with the higher high human development index, while more than a third due to imbalanced sugar and oil intake occurred in medium ones. Attributing weight-related risks to food intake can inform food-based interventions for reducing the burden of preventable deaths associated with imbalanced weight levels.

